# Ulerythema Ophryogenes in a Saudi Male: A Case Report

**DOI:** 10.7759/cureus.26593

**Published:** 2022-07-05

**Authors:** Nouf Algharbi, Yara Jazzar, Asem Shadid, Asem Almesfer

**Affiliations:** 1 Dermatology, King Faisal Specialist Hospital and Research Centre, Riyadh, SAU; 2 Dermatology, Alfaisal University College of Medicine, Riyadh, SAU; 3 Dermatology, State University of New York at Buffalo, Buffalo, USA; 4 Dermatology, King Fahad Medical City, Riyadh, SAU

**Keywords:** perifollicular erythema, keratosis pilaris, sos1 mutation, keratosis pilaris rubra atrophicans faciei, ulerythema ophryogenes

## Abstract

Ulerythema ophryogenes (UO) or keratosis pilaris rubra atrophicans faciei is a disorder of keratinization that primarily affects the face. The inflammatory process in UO may eventually result in alopecia. The incidence of this disorder is still unknown. We present a case of UO in a 28-year-old male, the first of its kind in Saudi Arabia.

## Introduction

Ulerythema ophryogenes (UO) is a rare dermatological disorder of keratinization primarily affecting the face. It manifests as erythema and multiple facial papules that result in atrophy, scarring, and alopecia [1]. A clear etiology is still unknown; however, several genetic syndromes have been linked to UO such as Noonan syndrome, Cornelia De Lange syndrome, Rubinstein-Taybi syndrome, and cardiofaciocutaneous (CFC) syndrome [2-5]. The term Ulerythema Ophryogenes was first introduced in 1889 by Tanzer [6], and in 1896, Unna used the term in his renowned book "The Histopathology of the Diseases of the Skin" [7]. Although the condition is benign, it remains not well understood which makes it worrisome. The incidence is sporadic in some cases and inherited in an autosomal dominant manner in others [1]. To our knowledge, there is no epidemiological data sufficient to establish the prevalence of UO. However, no cases have been reported before in our country. We present the first case of UO in Saudi Arabia: an adult male with no previous medical history.

## Case presentation

**Figure 1:**
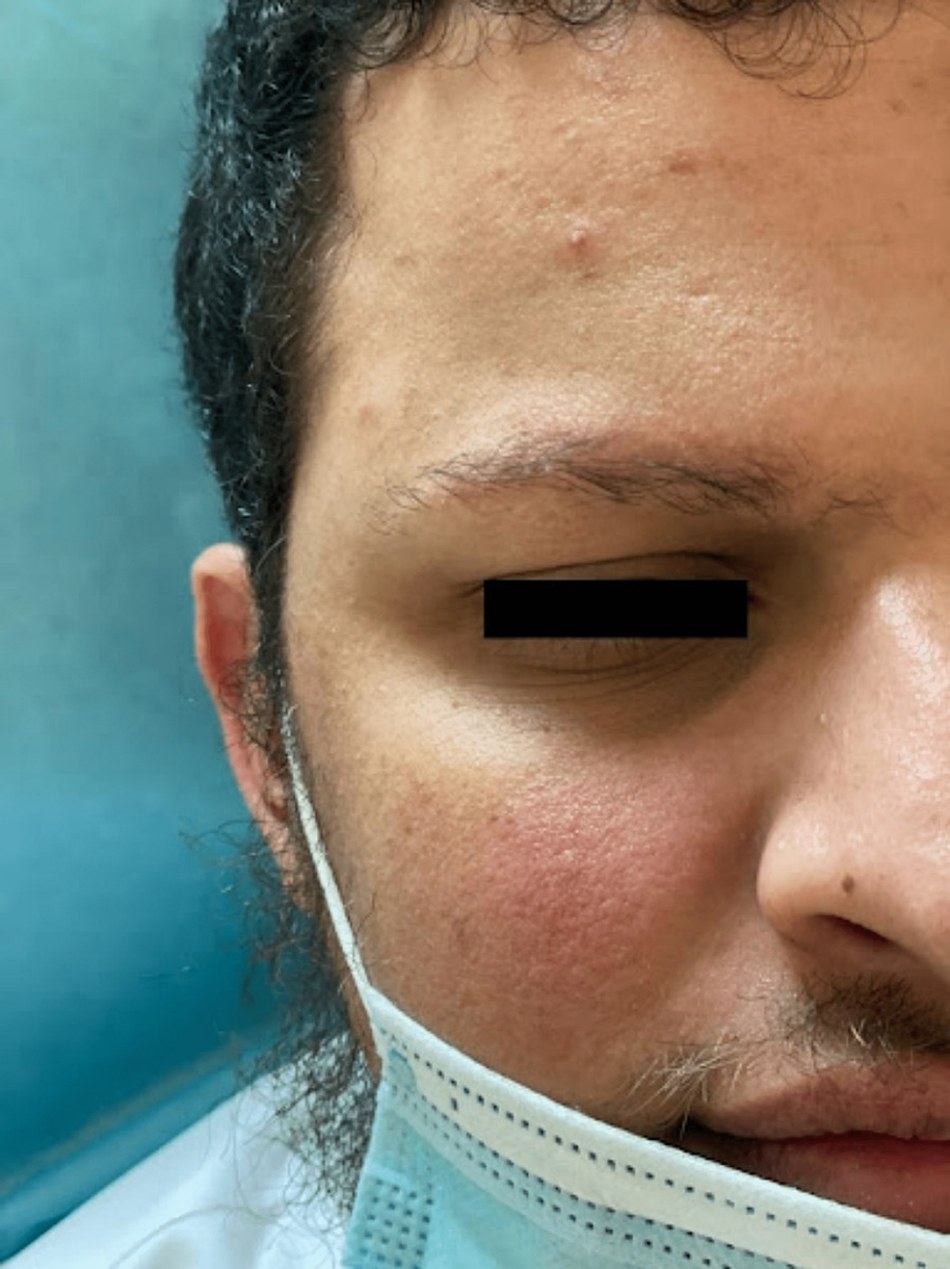
Prominent telangiectasia and macular erythema sparing the nasolabial folds on the right side of the face.

**Figure 2:**
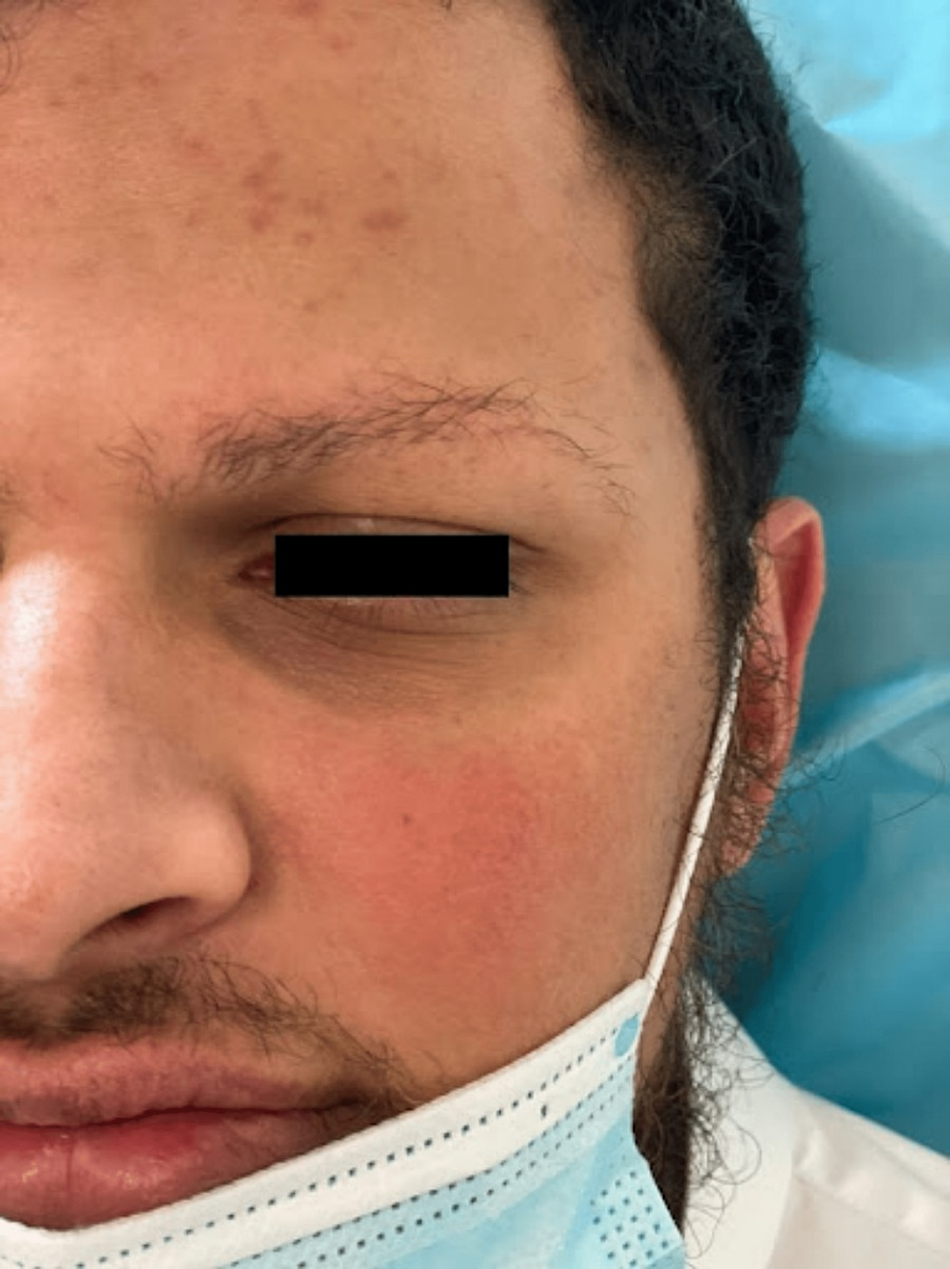
Prominent telangiectasia and macular erythema sparing the nasolabial folds on the left side of the face

**Figure 3:**
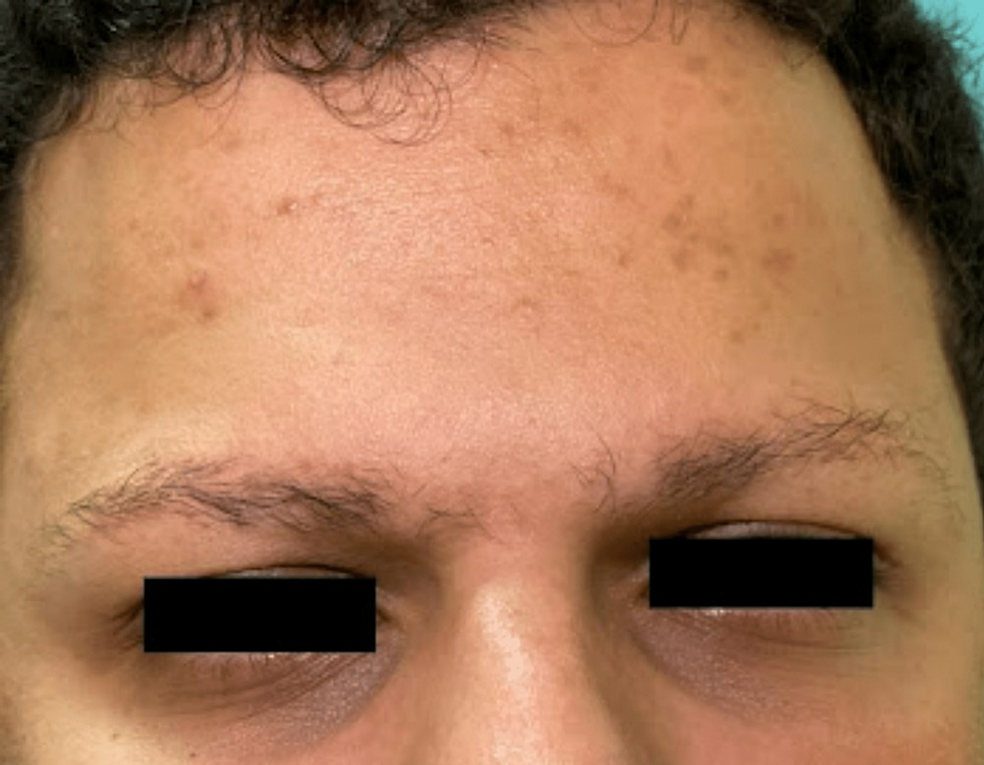
Multiple well-defined red to brown papules are present over the forehead. Eyebrows show areas of alopecia as well as well-defined confluent follicular erythematous papules.

We report a case of a 28-year-old Saudi single male who presented to our dermatology clinic for progressive asymptomatic facial erythema and gradual diffuse thinning of eyebrow hair that has been evident since childhood. He was seen by multiple dermatologists and was misdiagnosed on multiple occasions with alopecia areata (AA) but has never received any form of treatment. The patient's past medical history is insignificant, and his surgical history is only positive for cosmetic breast reduction surgery for gynecomastia. He had not been on any medications. His family history is negative for similar conditions including rosacea and AA. Cutaneous examination of the face revealed symmetrical well defined macular erythema with prominent telangiectasia over the cheeks sparing the nasolabial folds (Figures 1, 2), along with a few well-demarcated erythematous to brownish papules scattered over the forehead (Figure 3). Eyebrow examination showed well-defined confluent follicular erythematous papules with areas of alopecia and no signs of pitted or atrophic scars (Figure 3). No other areas of skin were involved. Scalp, nails, and mucous membranes examinations were unremarkable. Based on the clinical picture and lack of dermoscopic features supporting other diagnoses, we diagnosed the patient with UO. The nature of the disease and treatment options were discussed with him including hair transplantation which unfortunately is not available at our center. He was started on pimecrolimus 1% cream twice a day, advised to apply sunscreen and emollients frequently, and scheduled for regular follow-up visits. Unfortunately, the patient did not show up to any of his scheduled appointments.

 

## Discussion

UO, also known as Keratosis pilaris rubra atrophicans faciei, is a rare and underreported variant of keratosis pilaris. It is characterized by perifollicular erythema and horny red papules particularly involving cheeks, eyebrows, and the nasal bridge (malar area) [8]. It may also progress to involve the periauricular regions of the head and the scalp. These manifestations may eventually result in alopecia and scarring. An SOS1 mutation has been reported in a patient with Noonan syndrome and UO [4]. The mode of inheritance remains unknown; however, some studies have demonstrated an autosomal dominant pattern [2]. The treatment for UO is usually that of keratosis pilaris, conservative treatment. Patients are instructed to use sun protection, as the area affected is usually the sun-exposed skin [5]. The use of a soapless cleanser on a daily basis is also required to prevent excessive dryness [9]. Topical keratolytic agents can be applied twice daily and can be used interchangeably with low-potency corticosteroids such as triamcinolone acetonide (0.1%) [5], which is usually applied if the area is acutely inflamed once or twice daily for seven to ten days [9]. Tacrolimus ointment (0.1%) or white petroleum jelly has also been shown to improve keratosis pilaris [10]. As for laser and surgical treatments, recent studies have demonstrated the efficacy and safety of Pulsed Dye Laser (PDL) at a 585-nm or 595-nm wavelength [11]. In most cases, patients usually present during childhood years at the age of 5-16, with their guardian, as spontaneous resolution during adulthood is common [5].

## Conclusions

In conclusion, the incidence and prevalence of UO are still unavailable, and no ethnic, racial, or gender associations have been established. We report a case of UO in a 28-year-old male Saudi, the first of its kind in our country, Saudi Arabia, and even in the middle east region.
